# Airway smooth muscle NOX4 is upregulated and modulates ROS generation in COPD

**DOI:** 10.1186/s12931-016-0403-y

**Published:** 2016-07-19

**Authors:** Fay Hollins, Amanda Sutcliffe, Edith Gomez, Rachid Berair, Richard Russell, Cédric Szyndralewiez, Ruth Saunders, Christopher Brightling

**Affiliations:** Institute for Lung Health, Department of Infection, Immunity & Inflammation, Glenfield Hospital, University of Leicester, Leicester, LE3 9QP UK; Genkyotex, Geneva, Switzerland

**Keywords:** COPD, Oxidative stress, Reactive oxygen species, Airway smooth muscle, NOX4

## Abstract

The burden of oxidative stress is increased in chronic obstructive pulmonary disease (COPD). However, whether the intra-cellular mechanisms controlling the oxidant/anti-oxidant balance in structural airway cells such as airway smooth muscle in COPD is altered is unclear. We sought to determine whether the expression of the NADPH oxidase (NOX)-4 is increased in airway smooth muscle in COPD both in vivo and primary cells in vitro and its role in hydrogen peroxide-induced reactive oxygen species generation. We found that in vivo NOX4 expression was up-regulated in the airway smooth muscle bundle in COPD (*n* = 9) and healthy controls with >20 pack year history (*n* = 4) compared to control subjects without a significant smoking history (*n* = 6). In vitro NOX4 expression was increased in airway smooth muscle cells from subjects with COPD (*n* = 5) compared to asthma (*n* = 7) and upregulated following TNF-α stimulation. Hydrogen peroxide-induced reactive oxygen species generation by airway smooth muscle cells in COPD (*n* = 5) was comparable to healthy controls (*n* = 9) but lower than asthma (*n* = 5); and was markedly attenuated by NOX4 inhibition. Our findings demonstrate that NOX4 expression is increased in vivo and in vitro in COPD and although we did not observe an intrinsic increase in oxidant-induced reactive oxygen species generation in COPD, it was reduced markedly by NOX4 inhibition supporting a potential therapeutic role for NOX4 in COPD.

Chronic obstructive pulmonary disease (COPD) is an important cause of morbidity and mortality [1–3] with abnormalities in the oxidant/antioxidant balance being implicated in its pathogenesis [4]. Increased airway smooth muscle (ASM) mass is a feature of small airway disease and is related to airflow obstruction [5–7]. Previous work in our group has implicated increased nicotinamide adenine dinucleotide phosphate (NADPH)-oxidase type 4 (NOX4) expression in the ASM in the intrinsic ASM hyper-contractility observed in asthma [8]. Although oxidant/antioxidant imbalance is believed to be a key pathogenic factor in COPD [4, 9], little work has currently been performed to investigate what role, if any, NOX4 plays. Importantly, NOX4 expression is increased by both TGF-β and TNF-α, which are elevated in the ASM and/or serum of COPD subjects [10, 11] implicating a potential for increased NOX4 expression. Therefore, we hypothesized that NOX4 expression is increased in ASM from subjects with COPD resulting in enhanced reactive oxygen species (ROS) and oxidative stress.

Subjects with COPD and healthy controls (age-, sex- and smoking status-matched with normal lung function) and asthmatics as a disease control were recruited from Leicester, UK. The study was approved by the Leicestershire Ethics Committee and all patients gave their written informed consent.

Two-micrometer sections from bronchial submucosal specimens from subjects with COPD and healthy controls were stained with anti-NADPH oxidase 4 antibody (rabbit monoclonal, 11 μg/mL; Abcam, Cambridge, United Kingdom). Staining intensity above isotype control was measured in ASM bundles by thresholding using Cell^F software (Olympus, UK), where the thresholding levels set on Hue, Saturation and Intensity scales by an observer blinded to the subjects' clinical characteristics.

Pure ASM bundles were isolated from bronchoscopic samples and used at passages 2–6. Real-Time Reverse Transcription–Polymerase Chain Reaction was performed (SuperScript Vilo cDNA synthesis kit, Express SYBR GreenER qPCR Supermix Universal; Invitrogen). Relative quantification of NOX4 mRNA expression was done using the comparative 2^-∆∆Ct^ method and expressed as fold change as previously described [12], with amplification of 18S RNA as the internal normaliser gene (18 s forward primer ;h18SRNA.891 F:GTTGGTTTTCGGAACTGAGG, 18 s reverse primer ;h18SRNA.1090R:GCATCGTTTATGGTCGGAAC; NOX4 forward primer; hNox4.598 F:TGGCTGCCCATCTGGTGAATG, and NOX4 reverse primer; hNox4.878R:CAGCAGCCCTCCTGAAACATGC. ASM was stained with a NOX4 monoclonal Alexa Fluor 488 conjugated antibody (Abcam) with or without prior incubations with 50 ng/mL TNFα for 24 h. Intracellular reactive oxygen species (ROS) was assessed by incubating the ASM with 10 mM 5-(and-6)-chloromethyl-29,79-dichlorodihydrofluorescein diacetate (DCFDA) [8] in the presence or absence of the NOX4 inhibitor 10 μM (GKT137831 gift from Genkyotex).

Statistical analysis was performed using PRISM Version 6 (GraphPad, La Jolla, CA). Parametric data were presented as mean (SEM) and nonparametric data as median (interquartile range). Analysis between groups was by *t* tests or Mann–Whitney tests and across groups by ANOVA or repeated measures ANOVA for paired data with appropriate *post hoc* pairwise comparisons. Correlation by Spearman rank test. *P* < 0.05 was considered significant.

Representative photomicrographs illustrating NOX4 expression in the ASM are as shown (Fig. 1a). NOX4 ASM expression was increased in the subjects with COPD and the control subjects with a >20 pack year smoking history compared to the control subjects with ≤20 pack year history (21.5 ± 2.9 % and 25.7 ± 3.3 versus 5.9 ± 1.7 % respectively; *P* < 0.001; Fig. 1b). The intensity of NOX4 expression was positively correlated with smoking history in the group as a whole (r = 0.48; *P* = 0.037) and healthy controls (r = 0.72; *P* = 0.019), but not for the subjects with COPD (r = 0.11; *P* = 0.785; Fig. 1c).

**Fig. 1:**
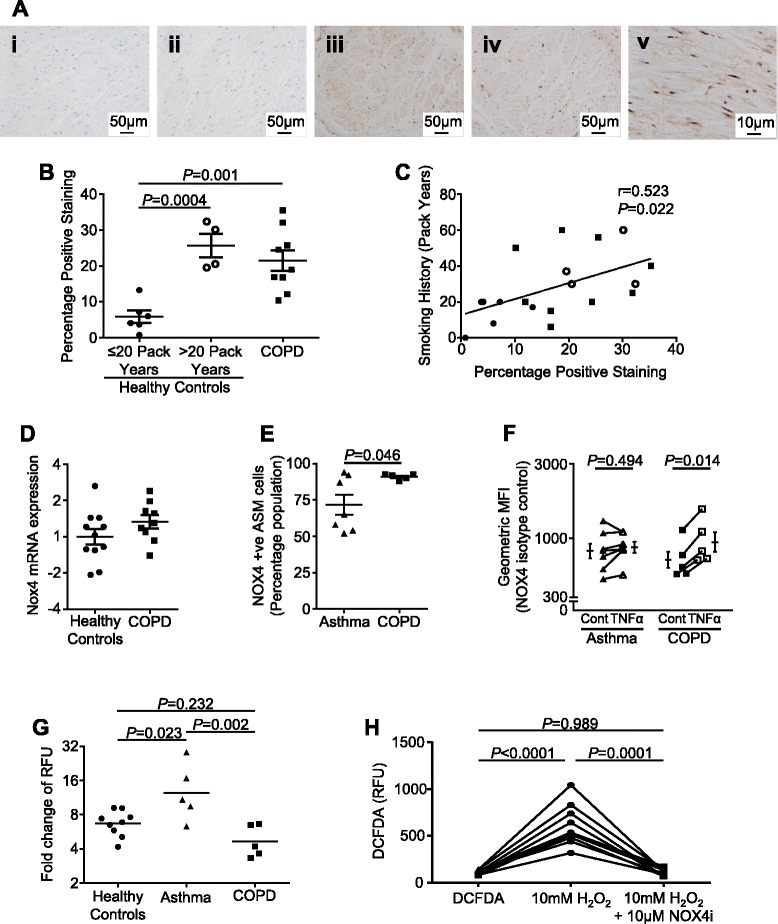
**a** Example photomicrographs of ASM stained with (i) isotype control or (ii-iv) NOX4 in a healthy control with a less than or greater than 20 pack year history and subject with COPD respectively and (v) an example of a COPD subject with more intense staining at higher power. **b** NOX4 % positive staining assessed by thresholding (mean [SEM]). *n* = 6 (<20 pack years healthy controls), *n* = 4 (>20 pack years healthy controls), *n* = 9 (COPD). **c** Correlation between smoking history and NOX4 ASM expression. **d** NOX4 mRNA (measured in triplicate) in ASM from COPD versus healthy controls. *n* = 11 (healthy controls), *n* = 9 (COPD). **e** Percentage of cells expressing NOX4 in ASM from COPD and asthma subjects assessed by flow cytometry. *n* = 7 (asthma), *n* = 5 (COPD). **f** Geometric mean fluorescence intensity of NOX4 expression following incubation with control media or 50 ng/mL TNFα in ASM from subjects with COPD or asthma. *n* = 7 (asthma), *n* = 5 (COPD). **g** Detection of intracellular ROS (measured in triplicate; relative fluorescent units [RFU]) in ASM induced by hydrogen peroxide. *n* = 9 (healthy controls), *n* = 5 (asthma), *n* = 5 (COPD). **h** Detection of hydrogen peroxide-induced intracellular ROS following pre-incubation with NOX4 inhibitor. *n* = 10. Each point represents an individual donor: circles-healthy, square-COPD and triangle-asthma subjects

NOX4 mRNA expression by primary cultured ASM from COPD subjects was not significantly different to either healthy controls (*P* = 0.174; Fig. 1d), nor our previously published NOX4 mRNA expression by ASM from asthmatic subjects (data not shown) [8]. We have reported that NOX4 protein expression as measured by flow cytometry was elevated in ASM from asthmatic compared to healthy subjects [8]. Here we show that the percentage of cells expressing NOX4 is significantly elevated in ASM from COPD versus asthmatic subjects (4of the asthma subjects were included in our previous report [8]) (91 ± 1 % versus 72 ± 7 % respectively; *P* = 0.046, Fig. 1e), although there was no difference in the geometric mean fluorescence intensity. Following treatment with 50 ng/ml TNFα for 24 h the geometric mean fluorescence intensity of NOX4 expression was significantly elevated in COPD (mean fold difference [95 % CI] 1.32 [1.22–1.43]; *P* < 0.001), but not asthmatic subjects (1.06-fold [0.93 to 1.21]; *P* = 0.29) (Fig. 1f).

ASM-derived from subjects with asthma, COPD and healthy controls were stimulated with hydrogen peroxide (10 mM). This concentration was chosen as a submaximal stimulus to promote ROS production without affecting cell viability. Following hydrogen peroxide exposure, there was an increase in the production of ROS by ASM from subjects with COPD, asthma and healthy controls (Fig. 1g). This increase was significantly greater in those with asthma compared to healthy controls and COPD, but was not different between COPD and health (Fig. 1g). This oxidant-induced ROS production was abrogated by the addition of NOX4 inhibitor (mean difference [95 % CI] -496.3 [−647.7 to −344.9]; *P* = 0.0001; Fig. 1h).

We show for the first time that in vivo NOX4 expression is up-regulated in ASM from COPD subjects and smokers with a >20 pack year history compared to smokers with a ≤20 pack year smoking history, and positively correlates to pack year history. We show that the percentage of NOX4 protein expression is significantly increased in primary ASM cells from COPD subjects compared to asthmatic subjects and that the intensity of expression is increased in COPD ASM following activation by TNFα. Oxidant-induced ROS generation in ASM from COPD subjects was similar to healthy control ASM and less than ASM-derived from asthmatic subjects. This oxidant-induced ASM ROS generation was completely abrogated by NOX4 inhibition in those subjects with or without COPD.

Oxidant/antioxidant imbalance is an important pathogenic factor in COPD [9]. This imbalance may be a consequence of environmental insults, the inflammatory milieu in COPD or due to abnormalities in the dynamic and complex processes involved in the generation and detoxification of ROS. Few studies have addressed the role of NOX4, Milara et al. showed that NOX4 expression and ROS generation are enhanced following exposure of human bronchial epithelial cells to cigarette smoke extract [13]. We have shown for the first time that in vivo NOX4 protein expression in ASM is elevated in COPD and controls with significant smoking histories. In addition we found that NOX4 protein expression in ASM cells from COPD subjects was further elevated compared to ASM from asthmatic subjects, and that the intensity of this expression in ASM from COPD subjects was enhanced in the presence of TNFα, which is increased in COPD [11].

Basal levels of ROS generation were not different between ASM from healthy control, asthmatic and COPD subjects. In contrast to the increase seen in asthma, the amount of ROS generated following stimulation with hydrogen peroxide using the DCFDA assay was similar in ASM from COPD subjects and healthy controls. Possible explanations for this apparent anomaly include differential NOX4 activity or activation/suppression of NOX4 independent pathways in the ASM derived from COPD subjects versus healthy controls. NOX4 plays an important role in the overall oxidative burden of the ASM, as demonstrated by our data showing that H_2_O_2_ stimulated ROS production in ASM can be completely abrogated with a NOX4 inhibitor. However the magnitude of this role in COPD needs to be further studied in vivo. It has previously been shown that NOX4 up-regulation and consequent ROS generation promote TGF-β1-induced proliferation and hypertrophy of ASM [14]. Whether up-regulation of NOX4 expression, and the oxidative burden in COPD in vivo contribute to ASM dysfunction in COPD subjects remains to be determined.

Thus, we provide evidence for up-regulation of NOX4 expression in vivo and in vitro in the ASM from COPD subjects and complete abrogation of oxidant-induced ROS generation by NOX4 inhibition. The development of NOX4 small molecule inhibitors in clinical trials for other disease indications (NCT02010242) means that the role of NOX4 in COPD could be tested in the clinic in the near future.

## Abbreviations

ASM, airway smooth muscle; COPD, chronic obstructive pulmonary disease; DCFDA, 5-(and-6)-chloromethyl-29,79-dichlorodihydrofluorescein diacetate; NOX4, nicotinamide adenine dinucleotide phosphate oxidase type 4; ROS, reactive oxygen species; TGFβ, transforming growth factor β; TNFα, tumour necrosis factor α
